# Applicability of the Global Lung Initiative 2022 Reference Equations on a Sample of Healthy Adolescents in Jordan

**DOI:** 10.3390/children13050613

**Published:** 2026-04-28

**Authors:** Walid Al-Qerem, Anan Jarab, Fawaz Alasmari, Alaa Hammad, Khalda Smairan, Judith Eberhardt

**Affiliations:** 1 Department of Pharmacy, Faculty of Pharmacy, Al-Zaytoonah University of Jordan, Amman 1173, Jordan; waleed.qirim@zuj.edu.jo (W.A.-Q.); alaa.hammad@zuj.edu.jo (A.H.); khaldahussen999@gmail.com (K.S.); 2 Department of Clinical Pharmacy, Faculty of Pharmacy, Jordan University of Science and Technology, Irbid 22110, Jordan; anan.jarab@aau.ac.ae; 3 Department of Pharmacology and Toxicology, College of Pharmacy, King Saud University, Riyadh 11451, Saudi Arabia; 4 Department of Psychology, School of Social Sciences, Humanities and Law, Teesside University, Borough Road, Middlesbrough TS1 3BX, UK; j.eberhardt@tees.ac.uk

**Keywords:** spirometry, reference equations, Global Lung Initiative, GLI-2022, adolescents, Jordan

## Abstract

**Background/Objectives**: The Global Lung Initiative (GLI) 2022 race-neutral spirometry reference equations were introduced to improve interpretability across populations; however, their performance in Middle Eastern adolescents remains insufficiently validated. This study evaluated the applicability of GLI-2022 among healthy Jordanian adolescents. **Methods**: Healthy adolescents were recruited from secondary schools across multiple Jordanian cities (July–November 2025). Spirometry was performed according to ATS/ERS standards using a single device and standardized procedures. GLI-2022 predicted values and z-scores were derived for forced expiratory volume in one second (FEV_1_), forced vital capacity (FVC), and FEV_1_/FVC. Calibration was assessed using mean (SD) z-scores and the proportion below the lower limit of normal (LLN; z < −1.645). Agreement between measured and predicted values was examined using Bland–Altman methods. LLN-based pattern classifications were compared with those obtained using the local reference equation and GLI-2012. **Results**: A total of 921 adolescents (482 males, 439 females; mean age 15.7–16.0 years) were included. GLI-2022 produced positive mean z-scores for FEV1 (0.51–0.73) and FVC (0.51–0.69), with low proportions below LLN for both indices (<2% in each sex), indicating underestimation of predicted lung volumes. Exact binomial testing confirmed that the observed proportions below LLN for FEV1 and FVC were significantly lower than the expected 5% in both sexes (all *p* < 0.001). The FEV1/FVC ratio showed smaller deviations (mean z 0.07–0.19), with 4.1% of females and 5.8% of males below LLN, and these proportions did not differ significantly from 5% (female *p* = 0.444; male *p* = 0.402). Mean observed-minus-predicted biases for FEV1 were +0.185 L in females and +0.306 L in males, and for FVC were +0.224 L and +0.351 L, respectively; FEV1/FVC bias was −0.15 percentage points in females and +0.60 percentage points in males. LLN-based pattern classification showed 98.7% overall agreement with the local equation and 99.7% with GLI-2012; concordance for obstructive and possible restrictive patterns was 93.5% and 100.0%, respectively. **Conclusions**: In healthy Jordanian adolescents, GLI-2022 appears to underestimate predicted FEV_1_ and FVC, yielding upward-shifted z-scores and fewer volume indices below LLN, while the ratio is less affected. Although LLN-based pattern classification was largely preserved, population-specific validation remains necessary before routine clinical adoption of GLI-2022 in Jordanian adolescents; extrapolation to other Middle Eastern adolescent populations should await additional regional validation.

## 1. Introduction

Asthma is the most frequently encountered pediatric chronic respiratory illness [1,2]. Global Asthma Network (GAN) Phase I data indicate that about one in ten school-aged children and adolescents worldwide had wheeze in the preceding 12 months, underscoring the substantial global burden of asthma symptoms in these age groups [3]. Severe asthma affects about 6.7% of adolescents worldwide, posing a substantial burden on their physical and psychosocial well-being [3]. In Jordan, around 2.38% of school-aged children and 6.2% of adolescents are diagnosed with asthma [4,5]. In order to build a comprehensive and effective treatment plan, it is essential to correctly diagnose and monitor asthma in this age group [6], and this process relies on the accurate interpretation of spirometry values [7]. However, several factors should be taken into account while interpreting spirometry measures, as they are dependent on age, gender, height, and ethnicity [8]. Additionally, the development of the respiratory tract continues throughout adolescence [9]. Therefore, spirometry results were compared to reference equations developed from individuals with similar biological characteristics, but many ethnic groups were under-represented [10].

As a result, the Global Lung Initiative (GLI) formulated multi-ethnic reference equations in 2012 (GLI-2012) that can be applied to a wide range of ages, from 3 to 95 years [11]. Yet, numerous subsequent validation studies found that these equations did not fully reflect the under-represented ethnicities, including children and adolescents from the Middle East [12], Africa [13], and China [14].

Consequently, in 2023, Bowerman et al. published the race-neutral spirometry equations designated as the GLI global (2022) equations; accordingly, the present study uses GLI-2022 throughout this manuscript to denote this equation set. These equations use age-, sex-, and height-based standards to estimate several spirometric measures. Nevertheless, the GLI-2022 equation was developed using data from only five broad ethnic groupings, namely Caucasians, African Americans, Mexican Americans, and Northeast and Southeast Asians [15]. Although the derivation cohort was substantially larger than those used for many earlier equations, external validation remains necessary in populations that were absent or underrepresented in the source data. This issue is particularly relevant during adolescence, when pubertal timing and rapid somatic growth may influence lung-function scaling.

Jordanian adolescents represent one such understudied population because no sex-stratified validation study has examined whether GLI-2022 is well calibrated in this setting or whether any miscalibration would alter interpretation relative to GLI-2012 or a locally derived Jordanian equation [16]. This study therefore addresses a clear gap in knowledge by testing whether a large race-neutral global equation performs similarly in an unrepresented national adolescent cohort. The study hypothesized that GLI-2022 would show incomplete calibration in Jordanian adolescents, particularly for volume indices, while preserving most LLN-based pattern classifications. Accordingly, the aim of the present study was to evaluate the applicability of GLI-2022 in Jordanian adolescents and to quantify its calibration, bias, and diagnostic concordance relative to GLI-2012 and the local Jordanian equation.

## 2. Methods

### 2.1. Study Population

Adolescents were recruited using a multistage, stratified, school-based random sampling design. Schools were sampled from the central, northern, and southern regions of Jordan, with sex and geographic region as the primary sampling strata. The initial target sample was 800 healthy adolescents, allocated equally by sex (400 males and 400 females). Regional allocation was proportional to population distribution, with target quotas per sex of 197 from Amman and the central region, 57 from Zarqa, 115 from the northern region, and 31 from the southern region. Within participating schools, classes were randomly selected, and eligible students were subsequently chosen by simple random sampling. Eligibility was determined using a health-screening questionnaire and spirometry quality assessment before inclusion in the analytical cohort. Recruitment continued within each sex-by-region stratum until the prespecified targets were achieved, with any necessary replacements restricted to the same stratum. Owing to strong participation, the final sample exceeded the initial target and comprised 921 adolescents (482 males and 439 females). Ethical approval was obtained from the Al-Zaytoonah University of Jordan Ethics Committee (Ref. No. 15/12/2024-2025), and the study was conducted in accordance with the Declaration of Helsinki.

Written parental consent and adolescent assent were obtained before spirometry. Inclusion criteria were enrollment in a selected secondary school, presence within the eligible adolescent age range, completion of the health-screening process, and ability to perform acceptable and reproducible spirometry according to the American Thoracic Society/European Respiratory Society (ATS/ERS) criteria [8]. To define the healthy reference population, adolescents were excluded if the screening questionnaire indicated asthma symptoms during the preceding 12 months, allergic rhinitis or hay fever, a recent respiratory infection, a smoking or vaping history, prematurity, or physician-diagnosed chronic lung disease beyond the symptom-based screen. The target sample size was based on the validation framework proposed by Quanjer et al. and was consistent with GLI-2012 Task Force recommendations, indicating that at least 150 males and 150 females are required to minimize sampling error when validating spirometry reference equations [11,17]. Participant flow is summarized in Supplementary Figure S1. In total, 1105 adolescents were screened for eligibility, of whom 124 were excluded before spirometry, including 92 who did not meet the healthy-participant criteria and 32 who lacked parental consent/assent or were absent on the testing day. Spirometry was performed in 981 adolescents. After quality control, 60 were excluded from the analytical dataset, including 55 with unacceptable maneuvers or failure to meet ATS/ERS reproducibility criteria and 5 with incomplete key data records. The final analysis therefore included 921 adolescents, exceeding the recommended minimum sample size in both sexes.

### 2.2. Anthropometric Assessment

To measure the height and weight of the children, a standardized electronic weighing machine and a stadiometer were used, and the results were rounded up to the nearest 0.1 kg and to the nearest centimeter.

### 2.3. Pulmonary Function Testing

Spirometry was performed in accordance with the ATS/ERS guidelines for pulmonary function testing [18]. Prior to testing, participants received standardized verbal instructions and a demonstration of the maneuver to ensure proper understanding and technique. All spirometry tests were performed using the same Minispir computerized spirometer (MIR, Rome, Italy) with original single-use flowMIR disposable turbines. These turbines are factory-calibrated and individually packaged, and the Minispir platform complies with ATS/ERS 2019 spirometry standards and ISO 26782/23747 requirements; the device is also FDA 510(k)-cleared [19].

Participants were tested in the seated position while wearing a nose clip to prevent nasal air leakage. Disposable turbines were used for each participant to maintain hygiene and reduce the risk of cross-contamination. Adolescents were instructed to inhale maximally to total lung capacity, followed by a rapid, forceful, and sustained exhalation for a minimum of six seconds or until an expiratory plateau was achieved. Each participant performed a minimum of three forced expiratory maneuvers. A maneuver was considered unacceptable if there was evidence of coughing during the first second of expiration, hesitation at the start of the maneuver, premature termination, variable effort, air leakage, or obstruction of the mouthpiece. Acceptability and reproducibility criteria were evaluated according to ATS/ERS recommendations. Reproducibility was defined as a difference of no more than 0.15 L between the two highest values of forced expiratory volume in one second (FEV_1_) and forced vital capacity (FVC). If reproducibility criteria were not met, additional attempts were performed, with a maximum of eight maneuvers allowed. Only spirometry results that met both acceptability and reproducibility criteria were included in the final analysis. The highest values of FEV_1_ and FVC obtained from acceptable maneuvers were used for data analysis, even if they were derived from different maneuvers.

### 2.4. Statistical Analysis

All analyses were performed using R (R Foundation for Statistical Computing, Vienna, Austria). Continuous variables were summarized as mean (standard deviation), and categorical variables were presented as frequency and percentage. Predicted values, percent predicted values, z-scores, and lower limits of normal (LLN) were derived for each participant using the GLI-2022 race-neutral reference equation and comparator equations (GLI-2012 and the local reference equation) for secondary analyses. The locally derived Jordanian pediatric spirometry reference equation was selected to be an important contextual comparator. This equation was developed from 1576 healthy Jordanian children aged 6–17 years, including 870 males, and modeled spirometric indices using generalised additive models for location, scale, and shape (GAMLSS) [16]. Because it was derived from the same national population and overlaps the age range of the present cohort, it provides a clinically relevant local benchmark for interpreting the performance of GLI-2022 in Jordanian adolescents.

LLN was defined using the conventional 5th percentile threshold (z < −1.645). For the primary validity assessment of GLI-2022, calibration was evaluated by (i) the mean and standard deviation of z-scores for FEV_1_, FVC, and FEV_1_/FVC within each sex, and (ii) the observed proportion below LLN for each parameter, reported with exact 95% binomial confidence intervals. Deviation from the expected 5% below LLN was assessed using exact binomial testing.

Agreement between observed spirometric measurements and GLI-2022 predicted values was further assessed using Bland–Altman methodology. For each sex, the mean bias (observed − predicted), root mean square error (RMSE), and 95% limits of agreement (mean bias ± 1.96 × SD of the differences) were computed for FEV_1_, FVC and FEV_1_/FVC. Bland–Altman plots were generated by plotting the observed–predicted difference against the mean of observed and predicted values, with the mean bias and limits of agreement superimposed for each stratum.

For comparative evaluation, paired within-subject differences in standardised values were calculated as Δz (z_GLI-2022 − z_Comparator) for each spirometric index, separately for GLI-2022 versus the local equation and GLI-2022 versus GLI-2012, and distributions were presented stratified by sex. Because these secondary between-equation analyses comprised 12 prespecified hypothesis tests (3 spirometric indices × 2 sexes × 2 equation contrasts), *p*-values for this family were adjusted using the Holm procedure [20]. Primary interpretation of model validity was based on effect sizes and interval estimates, including mean z-score centering, exact 95% confidence intervals for the proportion below LLN, and Bland–Altman bias and limits of agreement. To evaluate potential diagnostic impact, spirometric pattern classification was operationalised using LLN-based criteria. A reduced FEV_1_/FVC z-score (z < −1.645) was used to define an obstructive ventilatory impairment; cases with reduced FEV_1_/FVC and concurrently reduced FVC (z < −1.645) were classified as a potential mixed ventilatory impairment. Possible restrictive or nonspecific patterns were considered when FEV_1_/FVC was preserved (z ≥ −1.645) but FVC was reduced (z < −1.645), while normal spirometry was defined by z-scores above the LLN for both FEV_1_/FVC and FVC. Diagnostic shifts were quantified by cross-classifying GLI-2022-based categories against the comparator equations, overall and stratified by sex. All statistical tests were two-sided; for the 12 paired delta z comparisons, Holm-adjusted *p* < 0.05 was considered statistically significant.

## 3. Results

### 3.1. Sample Characteristics

A total of 921 adolescents (439 females) were included in the analysis. As presented in Table 1, comparable mean ages were reported between the sexes, with females having a mean age of 16.0 (1.44) years and males 15.7 (1.36) years. In contrast, anthropometric measures differed by sex, as males were both taller and heavier than females. Mean height was 166.1 (9.4) cm in males compared to 158.8 (6.0) cm in females, while mean weight was 58.6 (12.0) kg in males versus 55.9 (9.3) kg in females. Regarding spirometric measurements, males demonstrated higher absolute lung volumes, with greater mean values for FEV_1_ and FVC compared to females. Specifically, mean FEV_1_ was 3.74 (0.74) L in males and 3.06 (0.43) L in females, while mean FVC was 4.30 (0.91) L in males versus 3.42 (0.56) L in females. Conversely, the FEV_1_/FVC ratio was slightly higher in females (89.7 (5.9)%) than in males (87.5 (7.3)%).

### 3.2. Calibration of GLI-2022

The validity metrics of the GLI-2022 reference equation stratified by sex are summarized in Table 2. For both sexes, the mean z-scores for FEV1 and FVC were positive, indicating a systematic upward shift in observed values relative to GLI-2022 predictions. Mean z-scores for FEV1 were 0.51 (0.94) in females and 0.73 (1.06) in males, while for FVC they were 0.51 (1.08) and 0.69 (1.26), respectively. The proportion of participants with values below the lower limit of normal (LLN) was low for both FEV1 and FVC, remaining well below the expected 5% threshold in both sexes; exact binomial testing confirmed significant deviation from 5% for FEV1 and FVC in females and males (all *p* < 0.001). In contrast, the FEV1/FVC ratio showed a higher percentage below LLN, particularly in males (5.8%, 95% CI: 3.9–8.3) compared to females (4.1%, 95% CI: 2.4–6.4), and these proportions did not differ significantly from the expected 5% (female *p* = 0.444; male *p* = 0.402).

**Figure 1:**
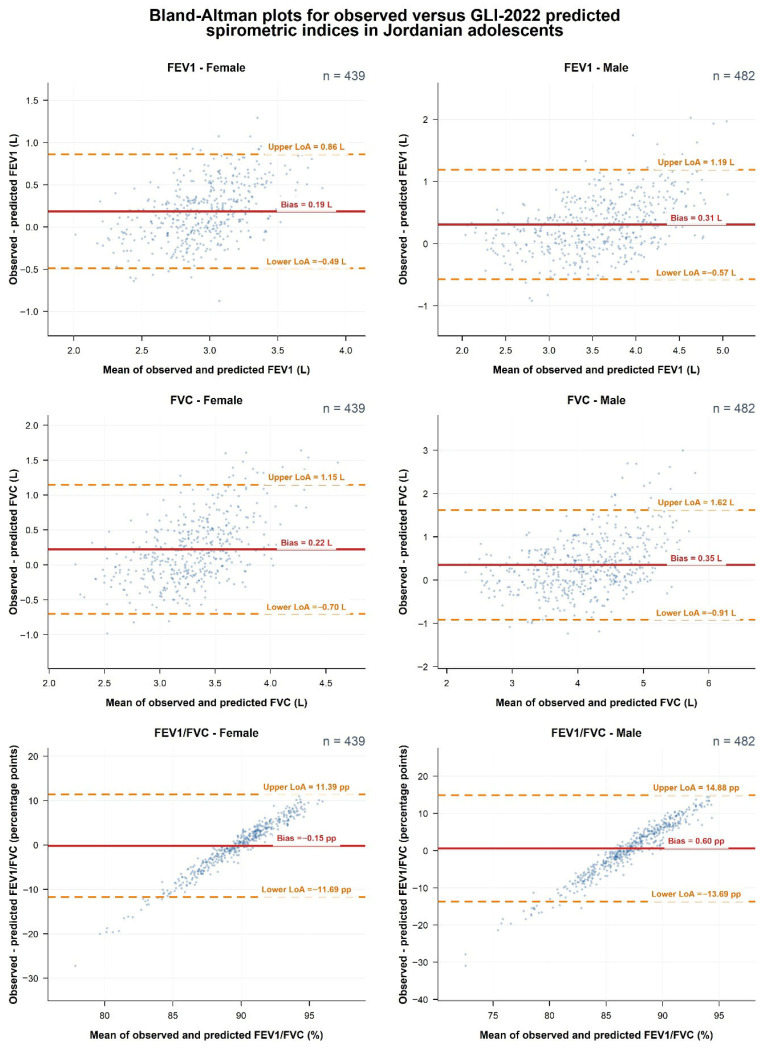
Bland–Altman agreement between observed spirometry and GLI-2022 predictions for FEV_1_, FVC, and FEV_1_/FVC.

Figure 1 presents Bland–Altman plots evaluating agreement between observed spirometric measurements and GLI-2022 predicted values for FEV_1_, FVC, and FEV_1_/FVC, stratified by sex, while Table 3 quantifies the corresponding observed-scale bias and precision. For both sexes, the volume indices showed positive mean bias, indicating that measured values generally exceeded GLI-2022 predictions, with larger discrepancies in males. For FEV1, mean bias was 0.185 L in females and 0.306 L in males, with RMSE values of 0.391 and 0.543 L and 95% limits of agreement of −0.490 to 0.860 L and −0.574 to 1.187 L, respectively. For FVC, mean bias was 0.224 L in females and 0.351 L in males, with RMSE values of 0.521 and 0.735 L and 95% limits of agreement of −0.700 to 1.148 L and −0.915 to 1.617 L, respectively. For FEV1/FVC, expressed in percentage points, bias was small in both sexes but differed in direction, at −0.15 in females and 0.60 in males; the corresponding RMSE values were 5.88 and 7.30, with limits of agreement of −11.69 to 11.39 and −13.69 to 14.88, respectively. Across all parameters, male adolescents showed wider RMSE values and limits of agreement, indicating greater variability in observed-predicted differences, although most observations remained within the 95% limits of agreement.

### 3.3. Equation Comparisons

**Figure 2:**
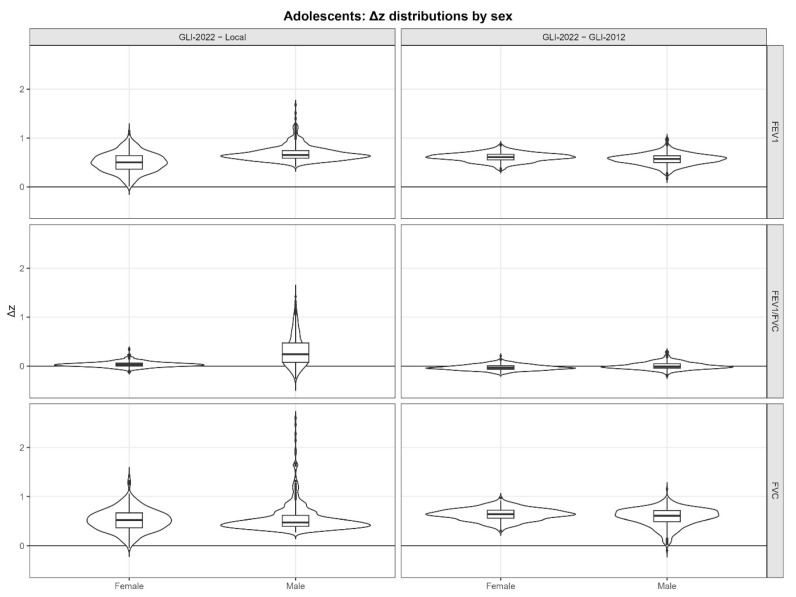
Paired differences in z-scores (Δz) comparing GLI-2022 with the local equation and GLI-2012, stratified by sex and spirometric index (FEV1, FVC, and FEV1/FVC).

The paired Δz distributions demonstrated a clear and systematic upward displacement of GLI-2022 relative to both comparator equations (Figure 2). For FEV1 and FVC, Δz values were uniformly positive in both contrasts (GLI-2022−Local and GLI-2022−GLI-2012), with medians consistently above zero and relatively compact interquartile ranges, indicating that GLI-2022 generally assigns higher standardized values than the local and GLI-2012 equations for the same individuals. This pattern was more pronounced in males, with a wider upper tail and occasional large positive deviations, suggesting greater between-equation divergence for a subset of male adolescents. By contrast, the FEV1/FVC ratio showed smaller and more heterogeneous differences: the GLI-2022−Local contrast was close to zero in females but shifted upward in males, whereas the GLI-2022−GLI-2012 contrast remained tightly centered near zero with only modest dispersion. After Holm adjustment for the 12 prespecified comparisons, all GLI-2022 versus local contrasts remained statistically significant (all adjusted *p* < 0.001), as did the GLI-2022 versus GLI-2012 contrasts for FEV1 and FVC in both sexes (all adjusted *p* < 0.001) and for FEV1/FVC in females (adjusted *p* < 0.001); the male FEV1/FVC contrast between GLI-2022 and GLI-2012 remained non-significant (adjusted *p* = 0.084).

Shifts in spirometric pattern classification when applying GLI-2022 compared with the local equation are presented in Table 4. Overall, agreement between the two approaches was high. Among participants classified as normal by the local equation, 98.9% remained normal under GLI-2022. Similarly, 93.5% of obstructive cases and 100% of possible restrictive patterns were consistently classified. Sex-stratified analysis revealed comparable findings, although males demonstrated a slightly higher degree of reclassification among obstructive cases compared to females.

When comparing GLI-2022 with GLI-2012 (Table 5), perfect concordance was observed for normal spirometric patterns, with all individuals classified as normal by GLI-2012 remaining normal under GLI-2022. Agreement was also high for obstructive and possible restrictive patterns, with only minimal discordance observed, indicating strong consistency between the two GLI equations in pattern classification.

## 4. Discussion

The present study evaluated the applicability of the GLI-2022 race-neutral spirometry reference equation in a large sample of healthy Jordanian adolescents and demonstrated a consistent upward shift in volume-based indices relative to GLI-2022 expectations. Both sexes showed positive mean z-scores for FEV1 and FVC, with a larger shift observed particularly in males. In parallel, the observed proportion below the LLN for FEV1 and FVC was markedly lower than the expected 5% for a well-calibrated reference in both sexes, and exact binomial testing confirmed that these deviations were statistically significant, indicating that GLI-2022 systematically underestimates predicted FEV1 and FVC in this population. In contrast, the FEV1/FVC ratio showed smaller deviations, particularly in females, with proportions below LLN that did not differ significantly from the expected 5% in either sex, suggesting that the principal calibration issue in this dataset is driven by lung volumes rather than the ratio.

The observed-scale analyses corroborate the z-score findings and provide clinically interpretable evidence that measured values exceeded GLI-2022 predictions, particularly among males [15]. The wider RMSE and Bland–Altman limits of agreement in males further suggest increased variability of the discrepancy in male adolescents, implying reduced precision of GLI-2022 predictions in this subgroup even when the direction of bias is consistent [21]. Taken together, these findings indicate that, in Jordanian adolescents, GLI-2022 tends to yield higher z-scores and larger positive observed-minus-predicted differences for FEV_1_ and FVC than would be expected under an ideally fitting reference, with the potential to reduce the apparent frequency of ‘low’ lung volumes in clinical interpretation.

The sex gradient observed in the present study is biologically plausible and aligns with known maturational heterogeneity during adolescence [9]. Pubertal timing and sex-specific trajectories of thoracic growth can produce transient deviations in spirometric scaling that are not fully captured by age and standing height alone, particularly in boys, where growth spurts may introduce additional variability and cohort-specific shifts [22]. Prior global work in school-aged children has highlighted that increased variance and age-spread effects in older pediatric groups may reflect pubertal influences, especially among boys, and that such effects can alter the fit of global equations across developmental stages [9,23]. In the current adolescent sample, the larger upward shift in males for FEV_1_ and FVC may therefore reflect a combination of population-specific growth patterns and differences in the maturational profile of the reference populations underlying GLI-2022. However, Tanner staging and other direct markers of pubertal status were not collected in the present study. Accordingly, this interpretation should be regarded as a hypothesis rather than a confirmed mechanism. Future studies incorporating pubertal staging, body composition, and ideally longitudinal follow-up are needed to determine whether maturational timing contributes to the larger positive shift observed in males.

Despite the continuous shifts in z-scores and measured-minus-predicted differences, the impact on spirometric pattern classification was limited. When GLI-2022 was compared with the local equation, agreement was high: among those classified as normal by the local equation, 98.9% remained normal under GLI-2022, while 93.5% of obstructive cases and 100% of possible restrictive patterns were concordant. Sex-stratified results were consistent with this overall pattern, although males showed a modestly higher degree of reclassification within obstructive cases. Similarly, comparison between GLI-2022 and GLI-2012 demonstrated perfect concordance for normal patterns and high agreement for obstructive and possible restrictive patterns, with only minimal discordance. This apparent stability of categorical diagnosis in the presence of a systematic volume shift suggests that, in this dataset, LLN boundaries for ratio- and volume-based criteria move in a way that preserves most pattern classifications, even though the underlying standardized distances from the reference distribution differ materially for FEV_1_ and FVC. Previous studies have shown that LLN-based spirometric pattern classification is relatively robust to systematic shifts in predicted volumes or z-scores, resulting in high diagnostic concordance even when reference equations differ [24].

The secondary comparative analyses further support that the primary divergence between GLI-2022 and the comparator equations is driven by volume outcomes. The paired Δz distributions indicated a systematic upward displacement of GLI-2022 relative to both the local and GLI-2012 equations for FEV_1_ and FVC, while differences for FEV_1_/FVC were smaller and more heterogeneous, particularly across sex. This pattern is consistent with the validity metrics in Table 2, where FEV_1_ and FVC showed clear positive mean z-scores and substantially depleted proportions below LLN, whereas the ratio was closer to the expected distribution, especially among females. From an interpretive perspective, this implies that equation choice in this adolescent population may have a larger impact on the perceived “normality” of lung volumes than on the diagnosis of obstruction based on the ratio alone, and that sex-specific effects should be anticipated when applying global reference standards in adolescent subgroups.

### Strengths, Limitations and Future Directions

Several limitations should be considered when interpreting these findings. First, spirometry can only suggest a possible restrictive pattern; confirmation of restriction requires lung volume measurements, which were not available in the present study. Second, pubertal stage and body composition measures were not collected; thus, the extent to which maturational timing explains the observed sex differences cannot be quantified. Third, although the inclusion criteria were designed to enrich healthy adolescents, residual confounding by unmeasured exposures (e.g., environmental pollution, passive smoking, or socioeconomic factors) may persist and could influence lung development and reference fit. Fourth, all spirometry measurements were obtained using a single Minispir computerized spirometer with original single-use flowMIR disposable turbines. This approach was intentional and minimized within-study inter-device heterogeneity. According to manufacturer specifications, the flowMIR turbines are factory-calibrated and individually packaged, and the Minispir platform complies with ATS/ERS and ISO spirometry standards [19]. Published pediatric and adolescent head-to-head studies have generally reported modest mean between-device differences for FEV1 and FVC under supervised testing, including 0.066 L and 0.053 L in adolescents aged 12–18 years and 0.079 L and 0.075 L in school-aged children [25,26], all of which are materially smaller than the systematic positive shift observed in the present cohort. Accordingly, although some contribution from platform-specific measurement characteristics cannot be excluded, device-related variation alone is highly unlikely to fully explain the pattern observed in the present study. Nonetheless, the study has notable strengths, including a large sex-stratified sample, standardized spirometry procedures, and evaluation of validity using complementary metrics (z-score centering, LLN frequency, observed-scale bias, and paired cross-equation comparisons), which together provide a coherent assessment of GLI-2022 performance in Jordanian adolescents.

In summary, GLI-2022 showed a systematic positive shift for FEV_1_ and FVC in Jordanian adolescents, particularly among males, indicating underestimation of predicted lung volumes and a substantially reduced proportion of values below LLN for volume-based indices. While spirometric pattern classification remained largely stable across equations, the magnitude and direction of the observed shift support cautious interpretation of GLI-2022 volume indices in Jordanian adolescents; whether similar findings extend to other Middle Eastern adolescent populations requires additional regional validation.

## Figures and Tables

**Table 1:** Characteristics of study participants.

Characteristic	Female (n = 439)	Male (n = 482)
Age (years)	16.00 (1.44)	15.70 (1.36)
Height (cm)	158.8 (6.0)	166.1 (9.4)
Weight (kg)	55.9 (9.3)	58.6 (12.0)
FEV1 (L)	3.06 (0.43)	3.74 (0.74)
FVC (L)	3.42 (0.56)	4.30 (0.91)
FEV1/FVC (%)	89.7 (5.9)	87.5 (7.3)

Note. Values are presented as mean (SD).

**Table 2:** GLI-2022 validity metrics stratified by sex.

Sex	Parameter	n	Mean z (SD)	% Below LLN (95% CI)
Female	FEV1	439	0.51 (0.94)	0.9 (0.2, 2.3)
Male	FEV1	482	0.73 (1.06)	0.6 (0.1, 1.8)
Female	FVC	439	0.51 (1.08)	0.7 (0.1, 2.0)
Male	FVC	482	0.69 (1.26)	1.5 (0.6, 3.0)
Female	FEV1/FVC	439	0.07 (0.99)	4.1 (2.4, 6.4)
Male	FEV1/FVC	482	0.19 (1.19)	5.8 (3.9, 8.3)

Note. LLN was defined as z < −1.645. Proportions are shown as % (95% exact binomial CI).

**Table 3:** Observed-scale bias and precision versus GLI-2022 predicted values in the study cohort.

Sex	Parameter	Unit	n	Mean Bias	RMSE	LoA
Female	FEV1	L	439	0.185	0.391	−0.490 to 0.860
Male	FEV1	L	482	0.306	0.543	−0.574 to 1.187
Female	FVC	L	439	0.224	0.521	−0.700 to 1.148
Male	FVC	L	482	0.351	0.735	−0.915 to 1.617
Female	FEV1/FVC	percentage points	439	−0.15	5.88	−11.69 to 11.39
Male	FEV1/FVC	percentage points	482	0.60	7.30	−13.69 to 14.88

Note. Bias is defined as observed minus predicted. FEV1 and FVC are reported in liters; FEV1/FVC bias is reported in percentage points. LoA denotes Bland–Altman 95% limits of agreement (mean bias ± 1.96 × SD of residuals).

**Table 4:** Cross-classification of spirometric patterns: local equation diagnosis (rows) versus GLI-2022 diagnosis (columns). Values are n (row %) *.

Local Equation Diagnosis (Rows)	GLI-2022: Normal	GLI-2022: Obstructive	GLI-2022: Possible Restrictive Pattern
Overall			
Normal	827 (98.9)	9 (1.1)	0 (0.0)
Obstructive	3 (6.5)	43 (93.5)	0 (0.0)
Possible restrictive pattern	0 (0.0)	0 (0.0)	39 (100.0)
Female			
Normal	398 (99.0)	4 (1.0)	0 (0.0)
Obstructive	0 (0.0)	18 (100.0)	0 (0.0)
Possible restrictive pattern	0 (0.0)	0 (0.0)	19 (100.0)
Male			
Normal	429 (98.8)	5 (1.2)	0 (0.0)
Obstructive	3 (10.7)	25 (89.3)	0 (0.0)
Possible restrictive pattern	0 (0.0)	0 (0.0)	20 (100.0)

* The mixed disorder category is omitted because no cases were identified.

**Table 5:** Cross-classification of spirometric patterns: GLI-2012 diagnosis (rows) versus GLI-2022 diagnosis (columns). Values are n (row %) *.

GLI-2012 Diagnosis (Rows)	GLI-2022: Normal	GLI-2022: Obstructive	GLI-2022: Possible Restrictive Pattern
Overall			
Normal	836 (100.0)	0 (0.0)	0 (0.0)
Obstructive	3 (6.5)	43 (93.5)	0 (0.0)
Possible restrictive pattern	0 (0.0)	0 (0.0)	39 (100.0)
Female			
Normal	402 (100.0)	0 (0.0)	0 (0.0)
Obstructive	1 (5.6)	17 (94.4)	0 (0.0)
Possible restrictive pattern	0 (0.0)	0 (0.0)	19 (100.0)
Male			
Normal	434 (100.0)	0 (0.0)	0 (0.0)
Obstructive	2 (7.1)	26 (92.9)	0 (0.0)
Possible restrictive pattern	0 (0.0)	0 (0.0)	20 (100.0)

* The mixed disorder category is omitted because no cases were identified.

## Data Availability

The data presented in this study are available on request from the corresponding author due to privacy, and ethical reasons.

## Supplementary Materials

The following supporting information can be downloaded at: https://www.mdpi.com/article/10.3390/children13050613/s1. Figure S1: Participant flow for the adolescent cohort.
