# Supplementary figures and images for: Applicability of the Global Lung Initiative 2022 Reference Equations on a Sample of Healthy Adolescents in Jordan
Source: Children (Basel). 2026 Apr 28;13(5):613. doi: 10.3390/children13050613 (PMC13204529; doi:10.3390/children13050613)

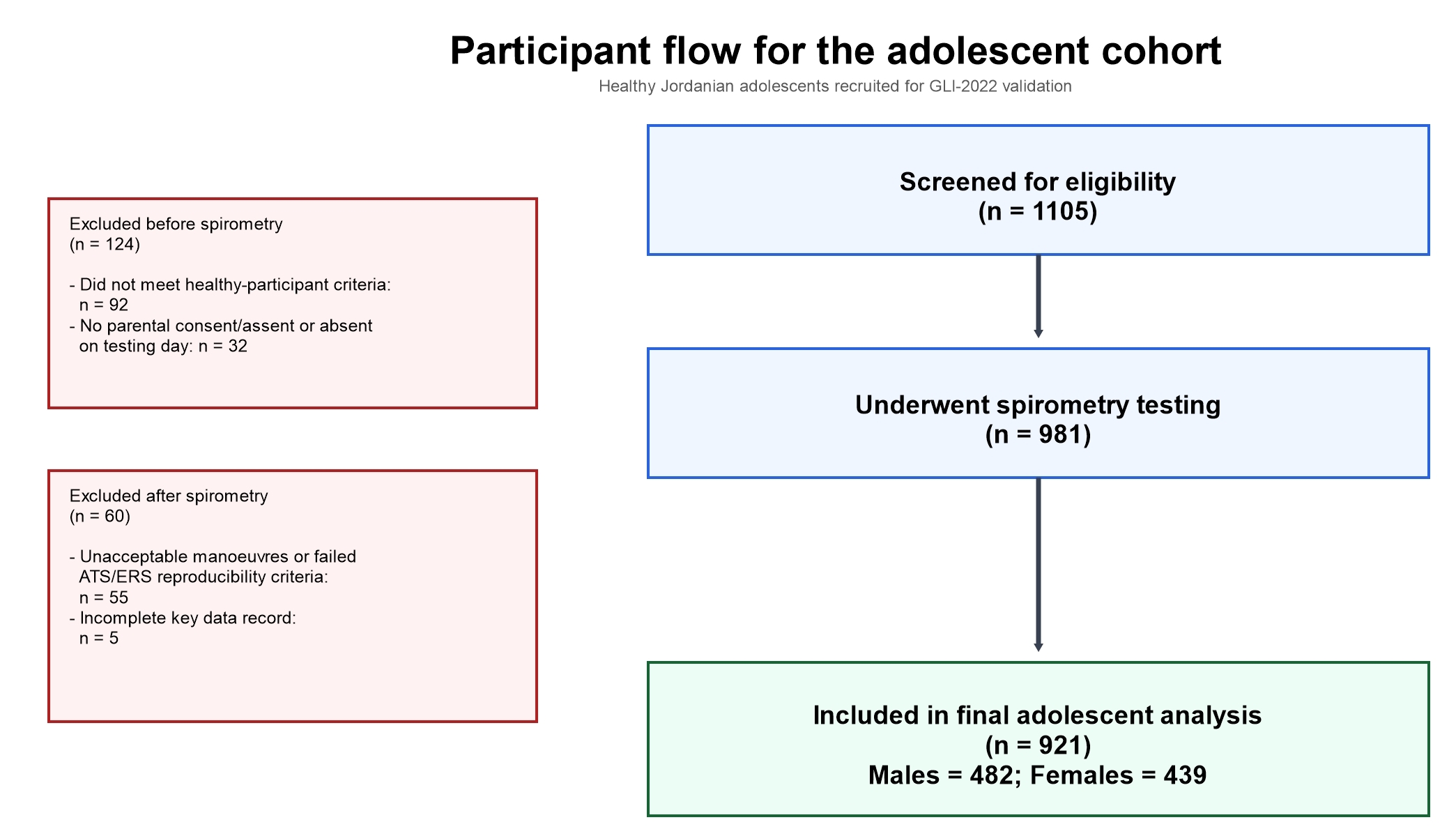

Supplement: Supplementary file 1 [file children-13-00613-s001.zip › children-4213721-supplementary.jpg]
